# Destabilization of the Outer and Inner Mitochondrial Membranes by Core and Linker Histones

**DOI:** 10.1371/journal.pone.0035357

**Published:** 2012-04-16

**Authors:** Annunziata Cascone, Celine Bruelle, Dan Lindholm, Paolo Bernardi, Ove Eriksson

**Affiliations:** 1 Research Program Unit, Biomedicum Helsinki 1, University of Helsinki, Helsinki, Finland; 2 Institute of Biomedicine, Biomedicum Helsinki 1, University of Helsinki, Helsinki, Finland; 3 Minerva Medical Research Institute, Biomedicum Helsinki 2, Helsinki, Finland; 4 Dipartimento di Scienze Biomediche Sperimentali, Università degli Studi di Padova, Padova, Italy; University of Medicine and Dentistry of New Jersey, United States of America

## Abstract

**Background:**

Extensive DNA damage leads to apoptosis. Histones play a central role in DNA damage sensing and may mediate signals of genotoxic damage to cytosolic effectors including mitochondria.

**Methodology/Principal Findings:**

We have investigated the effects of histones on mitochondrial function and membrane integrity. We demonstrate that both linker histone H1 and core histones H2A, H2B, H3, and H4 bind strongly to isolated mitochondria. All histones caused a rapid and massive release of the pro-apoptotic intermembrane space proteins cytochrome *c* and Smac/Diablo, indicating that they permeabilize the outer mitochondrial membrane. In addition, linker histone H1, but not core histones, permeabilized the inner membrane with a collapse of the membrane potential, release of pyridine nucleotides, and mitochondrial fragmentation.

**Conclusions:**

We conclude that histones destabilize the mitochondrial membranes, a mechanism that may convey genotoxic signals to mitochondria and promote apoptosis following DNA damage.

## Introduction

The genome is continuously exposed to external and internal genotoxic agents that can damage the DNA and lead to loss of genetic information. Damage to the genome activates the DNA damage response, a signal transduction pathway evolved to activate DNA repair mechanisms and to coordinate appropriate repair methods [1] or, in case of irreparable damage, to induce apoptosis [2]. Defects in the molecular switches between DNA repair and apoptosis are likely to contribute to degenerative disorders and tumor formation. However, DNA damage sensing and repair tailoring remains incompletely understood at molecular level.

Histones constitute the major protein component of chromatin. The first level of DNA compaction consists in the assembly of arrays of nucleosomes composed of two copies of each of the core histones H2A, H2B, H3 and H4, around which an about 147-bp long stretch of DNA is wrapped into nearly two superhelical turns. The linker histone H1 completes the DNA compaction, binding to the internucleosomal DNA near the entry and exit points of the nucleosome.

Core histones are highly conserved through evolution [3], [4] and they share a common fold domain consisting of three α-helices separated by loops [5]. The nucleosomes are stabilized by an extensive number of bonds between the amino acids of the histone fold domain and the phosphodiester backbone of DNA [6]. The histone fold domain is flanked by two tail regions which protrude from the nucleosome [5]. These tail regions undergo posttranslational covalent modifications which are of crucial importance for nucleosome stability and positioning along the DNA molecule. In contrast to core histones, linker histone H1 is less conserved through evolution [7] and has a distinct structure consisting of a short amino-terminal tail, a central winged-helix bundle, and a carboxy-terminal intrinsically disordered domain [8]. Linker histone H1 is subject to posttranslational covalent modifications that are important for chromatin compaction [9].

During the early DNA damage response the chromatin compaction around the damage site is relaxed through specific core histone modifications and by displacement of linker histone H1 [10]. The subsequent choice between repair and apoptosis may involve at least two histone-mediated switching mechanisms: (i) tyrosine phosphorylation of H2AX resulting in activation of the protein kinase JNK [11] and (ii) translocation of linker histone H1 from the nucleus to mitochondria [12], [13]. JNK1 is activated by a number of cell stress signals [14], leading to phosphorylation of several target proteins including the p53 tumor suppressor protein [15] and a subsequent activation of the mitochondrial apoptosis pathway [16], [17]. In contrast to H2AX, linker histone H1 appears to target directly the mitochondrial apoptosis pathway [12], [13], hence bypassing the transcriptional activation required for the JNK1 pathway.

The mitochondrial apoptosis pathway can be activated by intracellular stress signals and through receptor-mediated mechanisms [18]. Activation of the mitochondrial apoptosis pathway leads to a remodeling of the mitochondrial membrane architecture with an ensuing permeabilization of the outer membrane [19]. This event triggers the release of a set of pro-apoptotic proteins, including cytochrome *c*, from the mitochondrial intermembrane space to the cytosol [20], [21]. In the cytosol, these pro-apoptotic proteins activate caspases and endonucleases, leading to the cell's demise.

The discovery of linker histone H1-induced cytochrome *c* release [12] prompted us to study the interactions of both linker and core histones with mitochondria in order to address the mechanism and selectivity of cytochrome *c* release. To address these issues we employed a reconstituted system. We found that both core and linker histones bound strongly to mitochondria. This resulted in a rapid and massive release of cytochrome *c*, indicating that both linker histone H1 and core histones can permeabilize the outer mitochondrial membrane. However, unlike core histones, linker histone H1 permeabilized the inner membrane with a collapse of the membrane potential, release of pyridine nucleotides, and mitochondrial vesiculation. These findings demonstrate that both linker histone H1 and core histones have the capability to influence mitochondrial apoptosis signaling, and that histone H1 and core histones act on mitochondria through strikingly different molecular mechanisms. We propose a model for histone-mediated nucleus-to-mitochondria signaling in the setting of genotoxic DNA damage.

## Results

### Binding of linker and core histones to mitochondria

**Figure 1:**
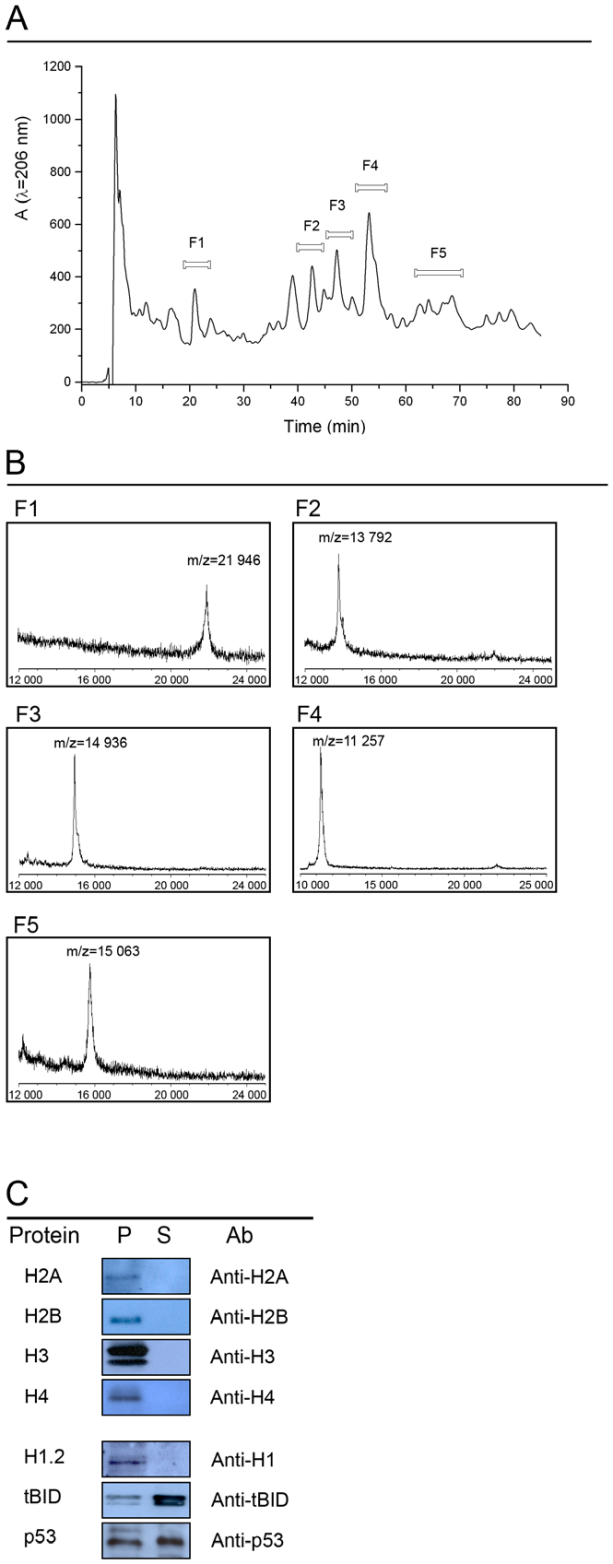
Purification of histones and binding to mitochondria. Panel *A*: histones extracted from rat liver nuclei by acid treatment were separated by reverse phase HPLC using a C18 column. All fractions were collected for analysis by MALDI TOF mass spectrometry and SDS-PAGE. The peaks labeled *F1* to *F5* were found to contain histones. Panel *B*: MALDI TOF mass spectra of compounds in fraction *F1* to *F5*. Fraction F1 contained histones H1.2; fraction F2, histone H2A; fraction F3, histone H2B; fraction F4, histone H4; and fraction F5 contained histone H3. Panel *C*: binding of histones, tBID, and p53 to mitochondria. Isolated rat liver mitochondria respiring on succinate as substrate were incubated with histones, tBID or p53. Mitochondria were sedimented by centrifugation and proteins of the resulting pellets (*P*) and supernatants (*S*) were analyzed by immunoblotting. Each protein was used at a concentration of 1 µM and the incubation was for 10 min at RT.

Linker and core histones were extracted from rat liver nuclei and purified to homogeneity by HPLC (Fig. 1A). The identity of each histone was verified by MALDI TOF mass spectrometry (Fig. 1B & Table 1) and by immunoblotting using anti-histone antibodies (data not shown). First, we investigated the binding of histones to mitochondria. Each histone (1 µM) was incubated with isolated rat liver mitochondria using succinate as substrate. After 10 min mitochondria were sedimented by centrifugation whereupon the resulting pellets and supernatants were analyzed by SDS-PAGE and immunoblotting. The results showed that histones were localized exclusively in the mitochondrial pellet (Fig. 1C) indicating that histones bind with high affinity to mitochondria. For comparison we investigated the pro-apoptotic proteins tBID and p53 [20], [16]. In contrast to histones, tBID and p53 bound only partially to mitochondria under these conditions (Fig. 1C).

**Table 1:** MS/MS analysis of peptides resulting from hydrolysis of the HPLC fractions by trypsin.

HPLC fraction	m/z	Amino acid sequence	Score	Hit
F1	1578.80	ALSSSGYDVEKNNSR	128	Histone H1.2
F2	944.54	AGLQFPVGR	75	Histone H2A
F3	1744.03	AMGIMNSFVNDIFER	61	Histone H2B
F4	1467.05	TVTAMDVVYALKR	86	Histone 4
F5	1055.75	EIAQDFTDLR	100	Histone 3

### Effects on mitochondrial membrane potential and ultrastructure

**Figure 2:**
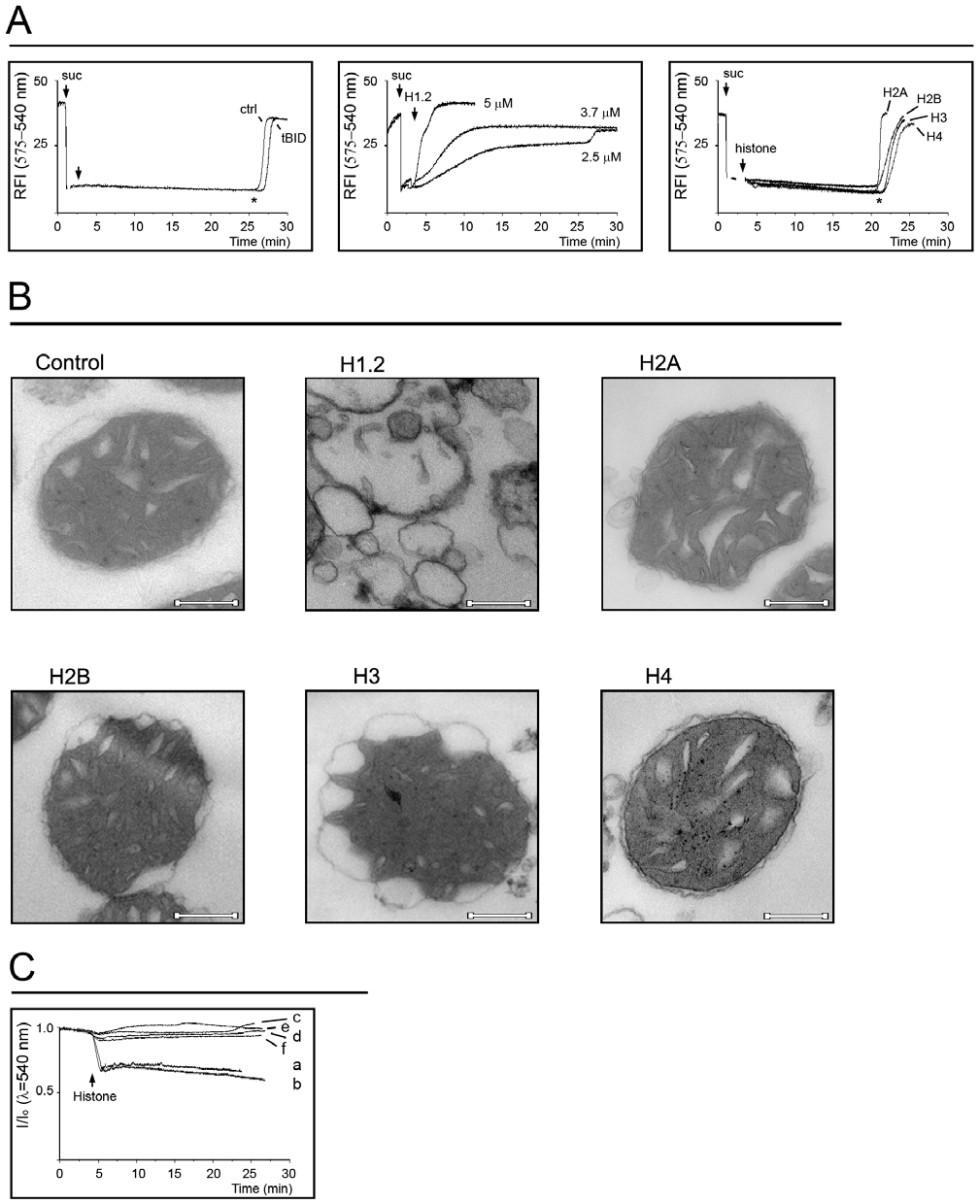
Effects of histones on mitochondrial function and ultrastructure. Panel *A*: mitochondrial membrane potential. Mitochondria were suspended in measurement medium supplemented with 0.5 µM TMRM. Succinate (*Suc*) was added to energize the mitochondria followed by 1 µM tBID (*tBID*), the indicated concentration of histone H1.2 (*H1.2*), 10 µM histone H2A (*H2A*), 10 µM histone H2B (*H2B*), 5 µM histone H3 (*H3*), or 5 µM histone H4 (*H4*). The *asterisk* indicates anoxia. Panel *B*: mitochondrial ultrastructure. Mitochondria suspended in measurement medium were incubated with histones for 30 min and then processed for transmission electron microscopy. The primary magnification is 50 000× and the scale bar is 200 nm. Panel C: light scattering. Mitochondria were suspended in measurement medium containing succinate. Histones were added as indicated. Trace *a* and *b*, 5 µM histone H1.2, in trace *b* the medium was supplemented with 1 µM cyclosporin A, trace *c* 10 µM histone H2A, trace *d*, 5 µM histone H3, trace *e*, 10 µM histone H2B, and trace *f*, 5 µM histone H4.

To investigate if histones influence basic mitochondrial functions we began by measuring membrane potential (ΔΨ) using the fluorescent probe tetramethylrhodamine methyl ester (TMRM). In the control experiment (Fig. 2A), addition of succinate to mitochondria led to a decrease in TMRM fluorescence due to ΔΨ-driven uptake of the probe into the mitochondrial matrix. After about 26 minutes the fluorescence intensity increased to initial level due to probe release indicating that the mitochondrial suspension had reached anoxia (Fig. 2A, left panel). Supplementing the suspension with tBID had no effect on the ΔΨ (Fig. 2A, left panel). Addition of histone H1.2 to respiring mitochondria induced a dramatic decrease in ΔΨ (Fig. 2A, middle panel). This effect was sharply concentration-dependent with a complete collapse of the ΔΨ within minutes after addition of 5 µM histone H1.2. In contrast to histone H1.2, addition of core histones had no significant effect on the ΔΨ or on the time required to reach anoxia (Fig. 2A, right panel).

Following these findings we examined the mitochondrial ultrastructure. Transmission electron microscopy (TEM) images of mitochondria prepared by chemical fixation are shown in Fig. 2B. Control mitochondria incubated without histones showed, as expected, a normal ultrastructure. However, addition of histone H1.2 resulted in striking effects including mitochondrial fragmentation and formation of vesicle aggregates, indicating that histone H1.2 caused a major disruption of the mitochondrial membrane architecture. In contrast to histone H1.2, core histones did not induce any major changes in the mitochondrial ultrastructure. However, in histone H3- and H4-treated mitochondria we frequently noted outer membrane blebs and ruptures (Fig. 2B).

Opening of the mitochondrial permeability transition pore (PTP) [22], a Ca^2+^-dependent megachannel, induces large-amplitude swelling and rupture of the outer membrane. We investigated if the ultrastructural changes induced by histone H1.2 were due to opening of the PTP measuring mitochondrial swelling by the decrease in light scattering of the mitochondrial suspension [23]. As shown (Fig. 2C, trace a) addition of histone H1.2 to mitochondria respiring on succinate resulted in a rapid decrease in the light scattering of the mitochondrial suspension. However, this decrease remained unchanged by the PTP inhibitor cyclosporin A (trace b) suggesting that the histone H1.2-induced decrease in light scattering was not due to PTP opening. Furthermore, cyclosporin A failed to prevent the effects of histone H1.2 on mitochondrial ultrastructure (data not shown). As predicted from the TEM results, core histones did not affect the mitochondrial light scattering (Fig. 2C, traces c to f).

### Release of mitochondrial pro-apoptotic proteins

**Figure 3:**
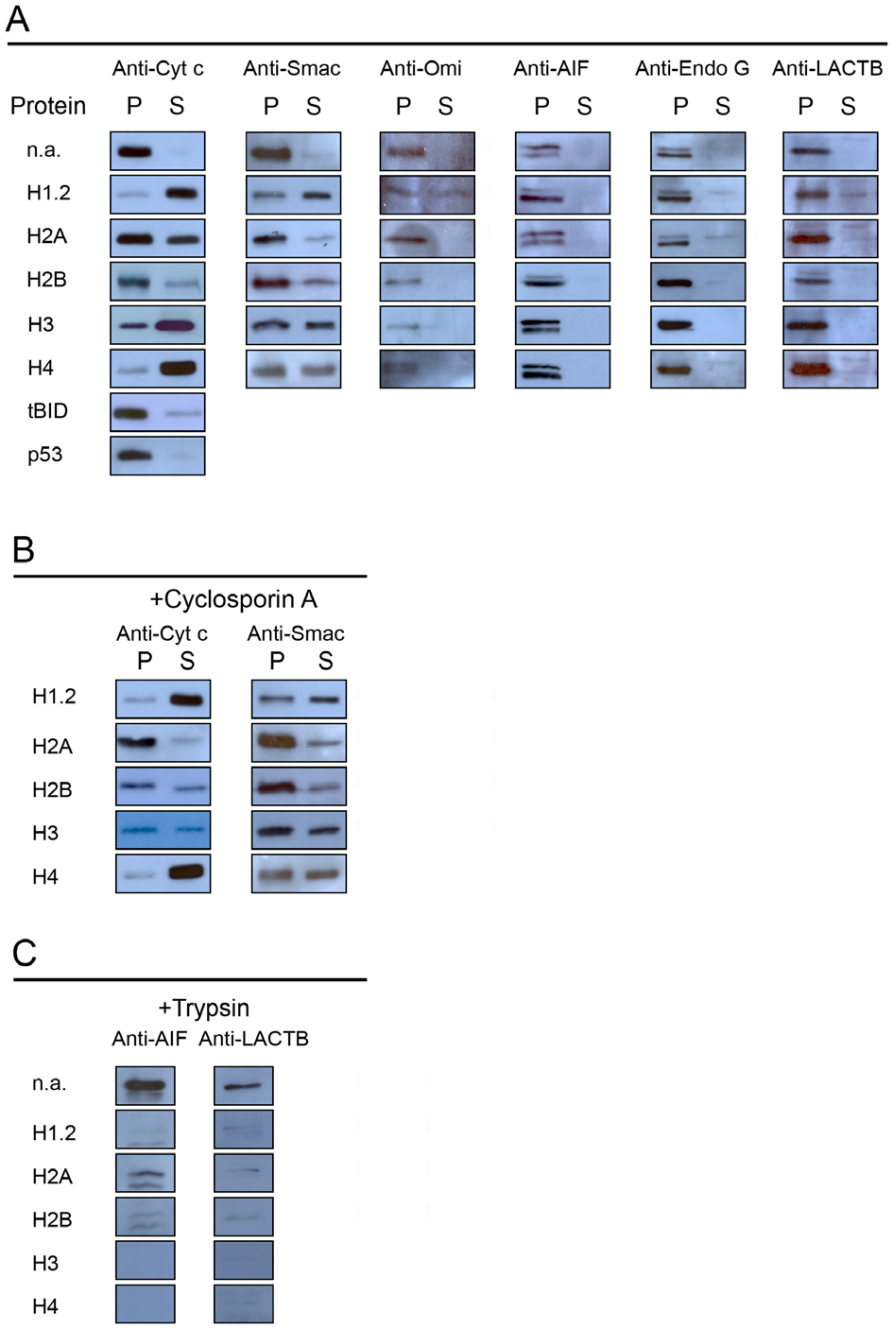
Release of mitochondrial intermembrane space proteins. Mitochondria were incubated as described in the legend of fig. 1, whereupon they were sedimented by centrifugation and proteins of the resulting pellets (*P*) and supernatants (*S*) were analyzed by immunoblotting. Panel *A*: release of cytochrome *c*, Smac/DIABLO, Omi/HtrA2, AIF, endonuclease G, and LACTB by histones, tBID and p53. The concentrations used were: 5 µM histone H1.2; 10 µM histone H2A, 10 µM histone H2B, 5 µM histone H3, 5 µM histone H4, 1 µM tBID, and 1 µM p53. Panel *B*: effect of cyclosporin A on the histone-induced release of cytochrome *c* and Smac/DIABLO. Panel *C*: accessibility of AIF and LACTB to trypsin hydrolysis in the presence of histones.

Mitochondria respiring on succinate as substrate were incubated with histones whereupon soluble proteins were separated from mitochondria by centrifugation. The resulting pellets and supernatants were analyzed by immunoblotting using anti-cytochrome c antibodies (Fig. 3A). In control mitochondria incubated without histones cytochrome *c* was recovered exclusively in the pellet. Upon addition of histone H1.2 a major part of the cytochrome *c* was recovered in the supernatant, in agreement with the findings of previous investigators [12], [13]. Unexpectedly however, after addition of core histones a substantial quantity of the total cytochrome *c* was recovered in the supernatant, histones H3 and H4 being most effective to release cytochrome *c*. In contrast to histones, addition of tBID or p53 resulted in a release of only a minor part of the total cytochrome *c*.

To determine if histones are capable of releasing other pro-apoptotic intermembrane space proteins we used antibodies against Smac/DIABLO [24], [25], the serine protease Omi/HtrA2 [26], the flavoprotein AIF [27], and endonuclease G [28]. We also included LACTB, a conserved protein forming high molecular weight filaments in the intermembrane space [29]. Our results (Fig. 3A) revealed that Smac/DIABLO was released both by linker histone H1.2 and by core histones. The extent of Smac/DIABLO release paralleled that of cytochrome *c*. In addition, histone H1.2 also induced a partial release of Omi/HtrA2, endonuclease G and LACTB, and promoted AIF cleavage. In contrast, these proteins were recovered almost exclusively from the pellet following incubation with core histones. The release of cytochrome *c* and Smac/DIABLO remained insensitive to cyclosporin A for all histones (Fig. 3B).

To investigate if histones induce a general increase in the permeability of the outer mitochondrial membrane we employed trypsin. Addition of trypsin alone to mitochondria respiring on succinate had no effect on Omi/HtrA2, AIF, endonuclease G, or LACTB as assessed by immunoblotting (Fig. 3C, data not shown for Omi/HtrA2 and endonuclease G), implying that the outer mitochondrial membrane remained intact. However, incubating mitochondria with core histones prior to trypsin addition rendered Omi/HtrA2, AIF, endonuclease G, and LACTB accessible to trypsin digestion, indicating that core histones permeabilize the outer mitochondrial membrane.

### Release of NAD^+^ from mitochondria

**Figure 4:**
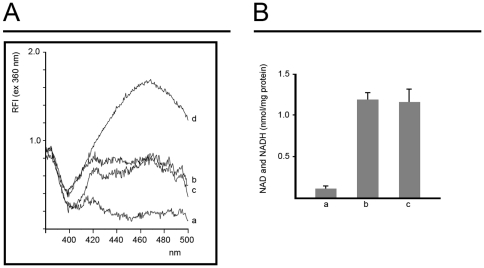
Release of pyridine nucleotides from mitochondria. Mitochondria were incubated as described in the legend of fig. 2, whereupon they were sedimented by centrifugation. The supernatants were collected for measurement of pyridine nucleotides by fluorescence spectroscopy after enzymatic conversion of NAD^+^ to NADH. Panel *A*: emission spectra of mitochondrial supernatants with the excitation wavelength set to 360 nm. Trace *a* shows control mitochondria. The mitochondrial suspension was supplemented with 50 µM Ca^2+^ in trace *b*, 5 µM histone H1.2 in trace *c*, and 1 µM NAD^+^ in trace *d*. Panel *B*: amounts of pyridine nucleotides released from mitochondria calculated from the fluorescence spectra. The experimental conditions were as in panel *A* (n = 4, error bars show S.E.M.).

Mitochondria harbor a large pool of NAD^+^, a compound which is required for DNA repair by poly(ADP-ribose) polymerase-1 (PARP-1) [30]. Therefore, we investigated if histones can induce release of NAD^+^ from mitochondria (Fig. 4). In the positive control we induced PTP opening through Ca^2+^ addition to induce inner membrane permeabilization and a complete release of mitochondrial pyridine nucleotides, as previously described [31]. NAD^+^ released from mitochondria was detected by fluorescence spectroscopy following enzymatic conversion to NADH (Fig. 4A). The results showed that following PTP opening 1.2 nmol NAD^+^/mg protein was detected in the mitochondrial supernatant (Fig. 4B). A similar quantity of pyridine nucleotides was released from mitochondria following addition of linker histone H1 (Fig. 4B) indicating that it had triggered a complete release of the mitochondrial NAD^+^ pool. In contrast, addition of core histones did not induce release of any detectable quantity of NAD^+^ above the control level (data not shown).

### Translocation of core histone H3 following DNA strand breaks

**Figure 5:**
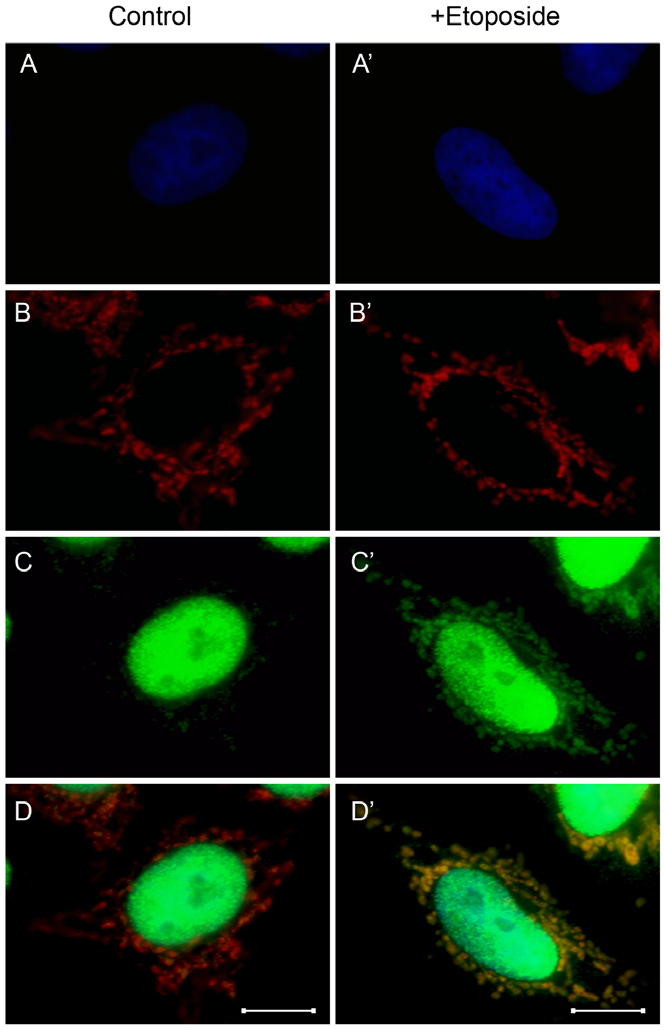
Translocation of histone H3 following DNA damage in mtRFP-HeLa cells. HeLa cells stably expressing mtRFP were incubated with etoposide and chemically fixed for immunofluorescence microscopy. *A* and *A′:* nuclei visualized with bisbenzimide staining (excitation 350 nm, emission 460 nm), *B* and *B′*: mitochondria visualized with mtRFP (excitation 553 nm, emission 574 nm), *C* and *C′*: anti-histone H3 antibody visualized with anti rabbit IgG Alexa 488 conjugate (excitation 485 nm, emission 531 nm), *D:* overlay of images *B* and *C*, and *D′:* overlay of images *B′* and *C′.* The scale bar is 5 µM.

We employed a HeLa cell line engineered to constitutively express red fluorescent protein targeted to mitochondria (mtRFP) as a model system to probe our findings in a cellular setting. DNA double strand breaks were induced by etoposide while monitoring the location of core histone H3 using the anti-histone H3 antibody (Fig. 5). Cellular nuclei stained with bisbenzimide are shown in panels *A* and *A′*, mitochondria harboring mtRFP are shown in panels *B* and *B′*, and core histone H3 visualized with a anti-IgG Alexa 488 is shown in panels *C* and *C′*. Overlay images between mitochondria and core histone H3 are shown in panels *D* and *D′*. The findings revealed that for control cells, core histone H3 remained confined to the nucleus, as evidenced by the distinct red mitochondrial fluorescence and green nuclear fluorescence. However, following induction of DNA strand breaks with etoposide, it is evident that a substantial fraction of core histone H3 has translocated from the nuclear compartment to the cytosol. Furthermore, the overlay image between core histone H3 and mitochondria reveal frequent yellow areas indicating co-localization of core histone H3 with mitochondria. These findings suggest that core histones have the capability to function as signaling molecules during DNA damage.

## Discussion

This study demonstrates that histones bind effectively to mitochondria, and that binding influences mitochondrial function and ultrastructure. Both linker and core histones induced a rapid and extensive release of the pro-apoptotic proteins cytochrome *c* and Smac/DIABLO. However, while the basic bioenergetic function of the inner membrane, i.e. the maintenance of a membrane potential, remained unaffected by core histones, linker histone H1.2 caused a disruption of the inner membrane resulting in loss of membrane potential, release of NAD^+^, and ultrastructural alterations. We conclude that both linker histone H1 and core histones have the capability to influence mitochondrial apoptosis signaling. However, the bioenergetic effects of linker histone H1 and core histones are very different which suggests that they act through separate molecular mechanisms, i.e. exclusively through outer membrane permeabilization for core histones, and through permeabilization of both the outer and inner membrane for linker histone H1.

Histones occur in massive quantities (60 million molecules per histone type) in nucleated cells. Therefore, the release of even a minor fraction of the total quantity of histones from the nucleus has the potential to impact on cell function. Nucleosomes are dynamic entities undergoing continuous restructuring with repositioning and unwrapping [32], [33]. DNA lesions promote nucleosome disassembly both through distortion of the DNA helix and through histone modifications [34]. A cell may withstand about 10^5^ DNA lesions per day under normal circumstances, but this figure becomes substantially higher after exposure to genotoxic agents [1]. A brief exposure to ionizing radiation causes a major perturbation in nucleosome organization with release of 5–10% of the total amount of histone H1 to the cytosol [12], resulting in an estimated cytosolic concentration at the order of 10^−4^ M. Viral infection can lead to massive nucleosome disassembly triggered by expression of viral DNA-binding proteins [35]. Our findings indicate that a histone concentration of 10^−6^–10^−5^ M is sufficient to cause dramatic effects on mitochondrial function. This raises the possibility that histones may be directly involved in nucleus-to-mitochondria signaling and that this function is not restricted to genotoxic stress events but extends to other forms of genome damage.

PARP-1 is highly expressed in the nucleus and plays a dual role during the DNA damage response. PARP-1 catalyses the transfer of poly(ADP-ribose) from NAD*^+^* to several nuclear target proteins [36]. During DNA damage repair the activity of PARP-1 increases substantially and this is of key importance for maintaining genome integrity. However, massive PARP-1 activation leads to cell death, possibly through depletion of the cytosolic and the mitochondrial NAD*^+^* pools, resulting in a bioenergetic crisis and a mobilization of mitochondrial pro-apoptotic proteins such as AIF [37]. However, the mechanism through which PARP-1 activation causes a depletion of the mitochondrial NAD^+^ has remained an enigma. We hypothesize that release of linker histone H1 may serve to mobilize the large mitochondrial pool of NAD*^+^* that is required for the PARP-1 reaction. Under conditions of limited DNA damage the action of linker histone H1 may be restricted to perinuclear mitochondria resulting in release of a quantum of NAD^+^ sufficient for the DNA-repair to take place, leaving peripherally located mitochondria intact and allowing for continuous ATP supply. However, under conditions of extensive DNA-damage a massive release of linker histone H1 may act on a majority of the mitochondria, resulting in a bioenergetic crisis and mobilization of mitochondrial pro-apoptotic factors. In this scenario, release of core histones would act in parallel to trigger a full-scale apoptotic response resulting in a rapid removal of the cells irreversibly committed to death.

Release of mitochondrial intermembrane space proteins can occur after opening of the mitochondrial PTP [22] and through outer membrane permeabilization by the BCL-2 protein pathway [37], [20]. In this setting, the action of linker histone H1 shares several key features with the PTP while, in contrast, core histones act in a similar way to the BCL-2 proteins. Therefore, it is tempting to speculate that linker histone H1 is acting, at least in part, through PTP opening and, likewise, that core histones are acting through the BCL-2 family protein pathway. However, the lack of effect of the PTP inhibitor cyclosporin A argues against an involvement of the PTP, although the possibility that linker histone H1 renders the PTP insensitive to cyclosporin A cannot be ruled out. Analogously, the finding that core histones were effective at releasing cytochrome *c* under these conditions, while the powerful BCL-2 pathway activator tBID was not, suggests that activation of the BCL-2 pathway is not necessary for selective outer membrane permeabilization, and that core histones act by a mechanism distinct from the BCL-2 protein pathway. We hypothesize that histones, due to their high positive charge density, interact primarily with cardiolipin (CL), a negatively charged phospholipid unique to mitochondria. An electrostatic mechanism may explain why the different core histones, sharing a common fold but little primary sequence similarity, exert similar effects on mitochondria. The organization and distribution of CL between the mitochondrial membranes influences the activity of a number of mitochondrial membrane proteins including components of the apoptosis machinery, and therefore, any perturbation in the CL dynamics can potentially impact on both mitochondrial function and ultrastructure [38].

Our findings underscore the existence of multiple molecular mechanisms for activation of the mitochondrial pathway to apoptosis. It is therefore possible that release of histones functions as an express route to trigger cell death under conditions of extensive DNA damage. Furthermore, these results demonstrate that cytochrome *c* can be released from mitochondria by physiological stimuli (i) through mechanisms not requiring major alterations in membrane architecture, as for core histones, or (ii) as a consequence of a disruption of the membrane architecture, as for linker histone H1. Therefore, the involvement of histone H1 in the cell death process, or the lack thereof, may provide a potential explanation to contradictory findings and views as to whether mitochondrial membrane remodeling is a prerequisite for, or consequence of cytochrome *c* release [39], [40].

## Materials and Methods

### Preparation of rat liver nuclei and mitochondria

Male Wistar rats (200–300 g) were killed by cervical dislocation under CO_2_ anaesthesia. The permit for use of laboratory rats was issued by the Helsinki University Laboratory Animal Center (permit number KEK11-011). The livers were excised and cut into pieces in isolation medium containing 250 mM sucrose, 10 mM Hepes-KOH, and 1 mM EGTA pH 7.4. The liver pieces were homogenized for 4 min in a Teflon potter (0.2 mm) maintained on ice-water mixture. The liver homogenate was filtered through cheesecloth and centrifugated at 800× *g* for 8 min. The pellets containing nuclei were collected and stored at −80°C. The supernatants were collected and mitochondria were isolated as previously described [23].

### Extraction of histones

Nuclear pellets from five rat livers were thawed, combined and supplemented with complete Protease Inhibitor Cocktail (Roche Diagnostics, Germany) according to manufacturer's instructions. The nuclear suspension was centrifugated at 10 000× *g* for 10 min and the resulting pellets were collected and divided into 10 ml aliquots which were suspended in 10 ml lysis medium containing 10 mM Tris-Cl pH 8.0, 1 mM KCl, 1.5 mM MgCl_2_, and 1 mM DTT supplemented with complete Protease Inhibitor Cocktail. The suspension was centrifugated at 10 000× *g* for 10 min. The resulting pellet was collected and resuspended in 10 ml lysis medium followed by centrifugation at 10 000× *g* for 10 min. The pellet was resuspended in 16 ml 0.2 M H_2_SO_4_ and mixed on a rotator for 3 h. The suspension was then centrifugated at 16 000× *g* for 10 min to remove insoluble material. The supernatant was collected and concentrated to a volume of 2 ml using a 3000 MWCO Amicon centrifugation filter (Millipore). The resulting 2 ml retenate was washed 6 times with 12 ml H_2_O in the same centrifugation filter. The retenate was then lyophilized and stored at −80°C. All extraction steps were carried out at +4°C.

### Purification of histones by HPLC

Histones were purified as described in [41] with the following modifications. Lyophilized nuclear protein extracts were dissolved in H_2_O. The solution was filtered through a 0.2 µm filter CA (FP30/0.2 µm Schleicher & Schuell) and then injected into a 100 µl HPLC sample loop. HPLC-UV analysis was carried out using an Äkta 10 HPLC system (Amersham Pharmacia Biotech, US) equipped with a P903 piston pump, a P900 UV detector, and a Frac-900 fraction collector using the UNICORN 5.10 software package. HPLC separation was performed using the conditions described by Naldi et al., 2006, with minor modifications. The column was a Phenomenex Jupiter 5U C18 300A 150×4.6 mm with SecurityGuard Cartridges Widepore C18 4×3.0 mm. Eluant A consisted of 0.04% heptafluorobutyric acid in H_2_O, and eluant B of 0.04% heptafluorobutyric acid in acetonitrile. The flow rate was 0.4 ml/min with a linear gradient from 38% B to 62% B lasting for 115 min. Proteins were detected at an absorption wavelength of λ = 206 nm. Fractions of interest were collected and concentrated to 1/3 of the initial volume (SpeedVac Concentrator, Savant). The fractions were then diluted with 3 volumes of H_2_O whereupon residual heptafluorobutyric acid was removed by repeated centrifugation through 3000 MWCO Amicon centrifugation filters (Millipore) until the pH was neutral. HPLC peaks were quantified using commercially available calf thymus histone H1 as standard. HPLC fractions were stored at a protein concentration of 100–200 µM at −80°C. A typical HPLC chromatogram is shown in Fig. 1A.

### Mass spectrometry

For analysis of protein masses samples containing about 1 µg protein were mixed with 1 µl of a saturated solution of sinapinic acid in 33% acetonitrile and 0.1% trifluoroacetic acid. The mixture was applied on the target plate and the solvent was allowed to evaporate. Matrix assisted laser-induced ion desorption and time-of-flight (MALDI TOF) analyses were performed using a Bruker Autoflex III mass spectrometer. Spectra were acquired with the linear detector operating in positive mode. Calibration was performed with bovine trypsin and horse cytochrome *c* as standard. Mass spectra of purified histones are shown in Fig. 1B. For analysis of peptide fragment masses samples containing about 10 µg protein were dried under vacuum and dissolved in 30 µl 10 mM ammonium phosphate pH 8.0, supplemented with 0.1 µg modified trypsin (Promega). The solution was incubated for 16 hours at +4°C whereupon the reaction was stopped by addition of 0.1% formic acid. Mass spectra of fragmented peptides deriving from the different histones are listed in Table 1.

### Mitochondrial functional measurements

Mitochondria were suspended at a concentration of 0.5 mg protein/ml in measurement medium containing 125 mM KCl, 10 mM Hepes-Tris, pH 7.4, 2 mM Pi, 100 µM EGTA, and 2 µM rotenone. Mitochondrial swelling was measured as the turbidity at λ = 540 nm. For measuring the mitochondrial membrane potential the suspension was supplemented with 0.5 µM of the fluorescent dye tetramethyl rhodamine (TMRM). The excitation wavelength was λ = 550 nm and emission was detected at λ = 575 nm. Turbidity and fluorescence was measured using a Cary Eclipse Fluorescence Spectrophotometer (Varian). Samples were removed from the fluorimeter cuvette and centrifugated at 10 000× *g* for 5 min. The resulting mitochondrial pellets and supernatants were used for immunoblotting and analysis of pyridine nucleotides. To measure binding of histones, tBID and p53 to mitochondria 1 µM of each protein was added to the mitochondrial suspension. After 10 min the suspension was centrifugated at 10 000× *g* for 5 min. The resulting pellet and supernatant were collected and used for immunoblotting. Functional measurements were performed at room temperature.

### Determination of pyridine nucleotides

To convert NAD**^+^** to NADH samples were supplemented with 22 mM ethanol and 0.1 U/ml alcohol dehydrogenase. NADH was quantified by fluorescence at an excitation wavelength of λ = 339 nm and an emission wavelength of λ = 460 nm using solutions with known NADH concentration as standard.

### Gel electrophoresis and immunoblotting

Mitochondrial pellets and supernatants were collected after incubations with histones, tBID, and p53. The supernatants were concentrated by centrifugation using Amicon (Millipore) ultracentrifugation filters with a molecular weight cut-off of 3 kDa. Mitochondrial pellets and concentrated mitochondrial supernatants were then resuspended to a final volume of 100 µl in H_2_O and solubilized by addition of 100 µl Laemmli gel sample buffer containing 125 mM Tris-HCl, 4% SDS, 20% glycerol, and 10% β-mercaptoethanol. Equal volumes of solubilized mitochondrial pelletes and supernatants were loaded onto 10% or 15% polyacrylamide gels. For each experimental condition used, mitochondrial pellets and supernatants were loaded onto the same gel. The running buffer was Tris/Glycine/SDS 10× (Bio-Rad). Gels were stained by Coomassie brilliant blue solution or used for electrotransfer onto a polyvinylidene fluoride membrane. Immuno-blotting was performed as previously described [29] and immunoreactive bands were visualized by chemoluminescence using the using the ECL system (Amersham Biosciences).

### Electron microscopy

Samples were removed from the fluorimeter cuvette and mixed with an equal volume of measurement medium supplemented with 6% DMSO and 5% glutaraldehyde. Samples were incubated for 2 min at room temperature and then centrifugated at 10 000× *g* for 10 min. The resulting pellet was detached from the centrifugation tube wall and incubated for 12 h at room temperature. Samples were prepared for transmission electron microscopy as previously described [29]. Thin-sections were viewed under a JEOL 1200 EX II electron microscope.

### Immunofluorescence microscopy

The HeLa cell line stably expressing mtRFP was generated as previously described [29]. Cells cultured on coverslips in Dulbecco's Modified Eagle's Medium supplemented with 10% fetal bovine serum were incubated with 100 µM etoposide dissolved in DMSO for 16 h to induce DNA strand breaks. Control cells were incubated with an equal amout of DMSO. Cells were then rinsed with PBS solution and chemically fixed with 4% paraformaldehyde for 20 min at room temperature. Cells were incubated with a rabbit polyclonal anti-histone H3 antibody diluted 1∶50 for 12 h at 4°C, followed by incubation with anti-rabbit IgG Alexa 488 conjugate diluted 1∶300 for 1 h at room temperature. The nuclear DNA was stained by incubation with bisbenzimide for 2 minutes at room temperature. Cells were then mounted and viewed under a Leica DM4500 B fluorescence microscope.

### Antibodies, recombinant proteins and chemicals

Polyclonal anti-histone H1 antibody, polyclonal anti-histone H2A antibody, and polyclonal anti-histone H2B antibody were from Santa-Cruz Biotechology, polyclonal anti-histone H3 antibody was from Cell Signaling Technology, polyclonal anti-histone H4 antibody, monoclonal anti-cytochrome *c* antibody, and polyclonal anti-AIF antibody were from Millipore, polyclonal anti-endonuclease G antibody was from Serotec, polyclonal anti-HtrA2/Omi antibody was from Alexis Biochemicals, polyclonal anti-Smac/DIABLO antibody was from Stressgen, polyclonal anti-LACTB antibody was prepared as previously described [29], anti-rabbit IgG peroxidase conjugate and anti-mouse IgG peroxidase conjugate were from Sigma, and anti-rabbit IgG Alexa 488 conjugate was from Invitrogen. Recombinant human wt p53 protein was from BD Pharmingen and recombinant human tBID was from Enzo Biosciences. Heptafluorobutyric acid was purchased from Fluka, histone H1 from Upstate, and TMRM from Molecular Probes. Organic solvents were purchased from Merck. Other chemicals and reagents were of highest grade and purchased from Sigma-Aldrich.
